# Hydrochlorate of Cocaine

**Published:** 1885-03

**Authors:** T. M. McIntosh

**Affiliations:** Thomasville, Ga.


### Correspondence.

### HYDROCHLORATE OF COCAINE.

### MORE CLINICAL EXPERIENCE WITH THE NEW ANÆSTHETIC—WHAT FAILURES IN ITS USES ARE DUE TO, ETC.

*Editors Atlanta Medical and Surgical Journal:*

Much has been written in regard to cocaine, and much that has been written has been of a very favorable nature. Occasionally failures are reported, for which the reason is found in deterioration from time, of the solution.

I have used the drug in several cases and of various kinds: fresh cuts, conjunctivas and torn sac operations, iridectomies, cataracts by discission, and in conjunctival tumors.

In all these I have obtained from partial to perfect anæsthesia, only upon the superficial surfaces. In none of my iridectomies has the pain of withdrawing and cutting the iris been lessened—certainly but very little.

The solution has been in every case of the same strength (four per cent.), prepared by E. R. Squibb, and the quantity has been from twenty to thirty drops in each case, using a few drops every two or three minutes during the lapse of from fifteen to thirty-five minutes. So there could be no fault with the quality of the preparation, the strength of the solution, or fear of its deterioration from age.

My very first case, the day upon which I received my preparation, was one of iridectomy for chronic irido-choroiditis, with complete circular synechia, marked increase of tension, associated with severe and constant pain. It did lessen the pain of speculum, forceps and incision, but certainly not the cutting of the iris, as the patient complained most violently, and had a nervous rigor. I was disappointed. Next day I applied it to a warty tumor on conjunctiva of lower lid, with perfect success.

In every instance it has done me good service on superficial parts; in every instance upon deeper operations it has failed me.

The cause of failures in operations on deep structures is due to its non-absorption, which might be overcome by introducing it to the required place with a hypodermic syringe, after first anæsthetizing the superficial parts.

It is destined, I think, to be a drug of great value in all minor surface operations, but I fear a more sanguine expectation will be disappointed, and in a wider range of application it will be found valueless.

Respectfully,

T. M. MCINTOSH.

*Thomasville, Ga., February 8th, 1885.*

